# Prescribed fire maintains host plants of a rare grassland butterfly

**DOI:** 10.1038/s41598-019-53400-1

**Published:** 2019-11-14

**Authors:** George C. Adamidis, Mark T. Swartz, Konstantina Zografou, Brent J. Sewall

**Affiliations:** 1 Department of Biology, 1900 North 12th St., Temple University, Philadelphia, PA 19122 USA; 2 The Pennsylvania Department of Military and Veterans Affairs, Fort Indiantown Gap National Guard Training Center, Annville, PA 17003 USA

**Keywords:** Fire ecology, Grassland ecology

## Abstract

As grassland ecosystems transform globally due to anthropogenic pressures, improvements in our understanding of the effect of management on rare and threatened species in such landscapes has become urgent. Although prescribed fire is a very efficient tool for habitat restoration and endangered species management on fire-adapted ecosystems, the specific mechanisms underlying potential effects of burning on population dynamics of butterfly host plants are poorly understood. We analyzed a 12-year dataset (2004–2015), combining violet abundance, habitat physiognomy and fire history data from a fire-managed system, to determine factors influencing the spatiotemporal distribution and abundance of violets (*Viola* spp.), the host plants of the threatened eastern regal fritillary (*Speyeria idalia idalia*) butterfly. Our results demonstrate a critical role for fire in driving both presence and abundance of violets, suggesting management with prescribed fires can effectively promote butterfly host plants. In addition, we determined the character of habitats associated with violet presence and abundance, in particular a strong positive association with biocrusts. These results provide a roadmap for efficient site selection to increase the effectiveness of restoration efforts, including assessment of potential reintroduction sites for regal fritillary and other grassland butterflies and actions to promote the re-establishment of host plants in these sites.

## Introduction

Temperate grasslands have undergone extensive change in the last century with profound implications for biodiversity. Globally, the temperate grassland biome has had the highest rate of conversion (>85% lost by 1990) and the least protection (1.9% in IUCN protected area categories I-IV) of any terrestrial biome^1^. Grassland remnants are also often highly fragmented^2^, and face critical ongoing threats from agricultural expansion, overgrazing, and invasion by exotic plant species (e.g.^3–8^). Such threats have severely impacted grassland biodiversity, including native plants (e.g.^9–11^) and insects^12^ such as grassland-specialist butterflies (e.g.^13–15^). Impacts to grassland biodiversity are often further exacerbated by anthropogenic suppression of wildland fires (e.g.^16^), without which many grasslands are ultimately replaced by forests or other biomes through succession^17^.

In recent years, managers have increasingly embraced prescribed fire to reinstate ecological processes formerly driven by wildland fires, while also reducing fuel loads and mitigating risk to people^18^. In this context, prescribed fire, although not a panacea, is arguably one of the most efficient tools for habitat restoration and endangered species management on fire-adapted grassland ecosystems^19^. For instance, populations of grassland butterflies often increase after prescribed fire^20,21^. This presumably occurs because fire indirectly benefits grassland butterflies by increasing the availability of nectar resources or the abundance, growth and reproductive output of host plants^20,22,23^, but the specific mechanism remains unclear. Clarification of this mechanism is important because, although plant and insect communities in grasslands have co-evolved with fire^24^, prescribed fires may also pose significant risks to habitat-restricted butterflies via direct mortality and loss of suitable habitat^25–27^. In particular, the adoption of prescribed fire often shifts the timing of peak fire seasons from the summer to the spring and fall months^18^ when some butterflies are in immobile or low mobility stages (egg, caterpillar, or pupa) and may be unable to escape the harmful effects of fire. Even if fire occurs in the adult stage when butterflies are more mobile, high fragmentation of a landscape may restrict access to suitable habitat until the burnt area recovers. In addition, given that butterflies are both key pollinators and herbivores throughout their life cycle, their host plants’ responses to prescribed burning could have significant cascading effects to ecosystem functioning. Thus, an improved understanding of mechanisms underlying the direct and indirect effects of prescribed fire on butterflies is needed^26,28^.

In this study we focused on violets, which are annual or perennial plants of a genus (*Viola*, Violaceae) that is relatively common, taxonomically diverse and has a wide geographic distribution. Many violet species have important dispersal/myrmecochory interactions with ants^29^ and serve as host plants for a variety of insect species (e.g.^14,30^). In North American grasslands, for instance, violets serve as the sole host plants for the regal fritillary (*Speyeria idalia* Drury), a rare North American butterfly that has experienced severe population declines throughout its range^14,31^, and is under review for listing under the United States Endangered Species Act^32^. *Speyeria idalia idalia*, the eastern regal fritillary butterfly, is a distinct subspecies of the regal fritillary^33,34^, whose sole population persists in a working landscape in south-central Pennsylvania, wholly within the borders of one of the busiest military training areas in the USA, Fort Indiantown Gap National Guard Training Center (hereafter FIG-NGTC). Since 2004, land managers at FIG-NGTC have used prescribed fire to maintain grassland habitat and benefit this imperiled butterfly. These actions appear to have had beneficial impact, leading to partial recovery in the eastern regal population^15^, presumably through an indirect pathway that includes a positive effect of fire on violets, though the specific mechanism remains unclear. An improved understanding of violet ecology could reveal more targeted and effective strategies to increase violet densities, promote recovery of the eastern regal fritillary and provide a roadmap for efficient facilitation of insect-host plant interactions on fire-managed systems.

Managers and researchers have also sought since 2011 to establish new populations of eastern regal fritillaries outside of FIG-NGTC that could reduce the likelihood of extinction, and enable recolonization if local extirpation does occur^35^. This effort, however, has yet to result in the establishment of a new viable population, which may imply low suitability of recipient habitats. Given that regal butterfly populations require sites with sufficient violets^14,36^, an understanding of violet ecology is crucial for accurately assessing the suitability of potential recipient habitats and ensuring the success of restoration efforts.

In this study, our aim was to determine factors influencing the spatial and temporal distribution and abundance of violets. Specifically, we first sought to determine to what extent fire is driving the local presence and abundance of violets. Second, as a means to predict habitat suitability of a site for violets in the absence of detailed fire history, we also sought to determine which biotic and abiotic factors are most associated with the local presence and abundance of violets. We combined extensive monitoring data on violets and biotic and abiotic characteristics of sites, precise local fire history data, and rigorous statistical modelling approach to clarify how prescribed fire management affects the presence and abundance of the host plants of the last viable population of a rare butterfly within a fire-managed grassland ecosystem.

## Materials and Methods

### Study sites

The study area was FIG-NGTC (40°26′13.15″N, 76°34′33.8″W), a military training area on the Appalachian Plateau physiographic region in Pennsylvania, USA. The landscape of the study area consists of a mosaic of forests and semi-natural grasslands extending over 6920 ha with altitudes ranging from 180 to 241 m^37^. The climate is humid continental with February the driest and May the most humid month. Our study was conducted at five distinct management units serving as study sites (B12, C4, D1, D3 and R23; Table S1) within grassland habitats at FIG-NGTC. All five sites currently host the entire remnant population of the extremely rare *S. i. idalia* butterfly^15,38^. We assigned sampling points throughout sites using a random-point generator in ArcView GIS 3.2 (ArcView 3.2 ESRI GIS and Mapping Software, Redlands CA) and we randomly generated one point per 0.40 ha for each site, resulting in a total of 265 sampling points.

### Prescribed fire

State biologists have since 2004 maintained a program for prescribed fire management within grasslands in FIG-NGTC^34^. This program is designed to meet several goals, including a reduction in the frequency and severity of wildland fires through the maintenance of early-successional plant community structure^19^, providing landscapes suitable for training with military vehicles, maintaining troop access to training landscapes, controlling the invasion of undesirable woody species (e.g.^39,40^) and management of populations of rare and threatened species (e.g.^41,42^) including the eastern regal fritillary. Study sites were managed with rotational burning during spring (March-May; hereafter spring fires) and late fall/winter (November-February; hereafter winter fires) according to a pre-determined fire rotation, with plans adjusted by vegetation growth, weather conditions, and fuel accumulation data. This versatile rotational burning, along with the fact that portions of each site are left unburnt in any given season and/or year, results in habitat patches with heterogeneous fire history.

### Violet sampling and environmental/vegetation measurements

Systematic surveys for violets abundance in the five study sites at FIG-NGTC were conducted in five years (2004, 2007, 2011, 2012 and 2015) during a 12-year period (2004–2015). In total, 995 surveys were conducted at the 265 plots located at random sampling points. In each survey, we placed a 2 m × 2 m quadrat, which provided a 4 m^2^ study plot, within which the total number of violet individuals was recorded. We found very few individuals of species other than *Viola sagittata* Ait. (Arrow-leaved Violet), which is the main eastern regal fritillary larval host species at FIG-NGTC sites^43^. However, we counted all violet species because regal fritillary larvae consume multiple species of violets^44^.

To facilitate evaluation of biotic and abiotic factors associated with violets, we also recorded six vegetation and two environmental variables within the same 4 m^2^ plots during all surveys after 2004. In particular, % cover of dead standing biomass, biological soil crusts (hereafter “biocrusts”; i.e. soil communities composed of moss, lichens, eukaryotic algae, cyanobacteria, liverworts and fungi^45^), warm-season grasses (e.g. *Schizachyrium scoparium* and *Andropogon virginicus*), cool-season grasses (e.g. *Fescue spp*., *Dichanthelium clandestinum*, *Agrostis gigantea*), woody plant litter (twigs and other plant parts produced by the managed thinning of woody plant species) and other vegetation (plant species not belonging to one of the previous categories) were visually estimated in each study plot and together these measurements represented the biotic character of our plots. The abiotic character of each plot was described by visually estimating % cover of bare ground and rocks. The recording of the biotic and abiotic factors was non-exclusive and due to overgrowth, the total of biotic and abiotic coverages may exceed 100%.

### Datasets

The fire history of the study plots at FIG-NGTC, including the location, spatial extent, and timing of both wildland fires and prescribed fires, is known precisely beginning January 1^st^ 1997 (pers. comm., forestry staff, FIG-NTGC), and so this date was used as the starting point to calculate the fire history of our sampling plots. Specifically, at each violet sampling, we calculated post-fire age (i.e. days since last fire or since January 1^st^, 1997) and fire frequency (number of times burned) for all our sampling plots.

Two overlapping datasets were developed, one for each objective of this study. First, to investigate the response of violets to prescribed fire, we used plot data from all five sampling years but excluded the 11.7% of plots that never supported violets, under the assumption that such plots, for reasons independent of fire management, did not provide a suitable substrate for violet growth and reproduction. Excluding these plots resulted in a dataset (hereafter Dataset1) with 879 plots.

Second, to investigate violet response to biotic and abiotic factors, we created a second dataset (hereafter Dataset2) incorporating plot data from only the four sampling years for which environmental and vegetation measurements were taken (i.e., 2007, 2011, 2012 and 2015). In Dataset2, we retained plots that never supported violets, to enable a comparison of non-suitable and suitable substrates for violet growth and thereby increase the predictive power of our model. Dataset2 consisted of 729 plots.

### Data analysis

All statistical analyses were performed using R version 3.4.2^46^. Even after removal of plots where violets were never observed from Dataset1, there was still a high percentage of violet observations equal to zero in a given year (33.1% of surveys were zeros). Dataset2 also had a high percentage of violet observations equal to zero (43.6% of surveys). Thus, zero inflation was present in both datasets. Given the sessile nature of violets, the small size of plots, and the exhaustive nature of surveys for violets within each plot, we considered zeros to represent plots where violets were not present (‘true’ zeroes), rather than plots where we failed to record existing violets (i.e. ‘false’ zeros; sensu^47,48^). Consequently, to address our scientific questions we employed zero-altered models, also referred to as hurdle models, since such models assume zeros are ‘true’^49^. Further, in line with the specific goals of this study, hurdle models enable simultaneous, distinct evaluation both of processes resulting in the presence (or absence) of host plants and of processes resulting in the abundance of host plants where they are present^49^.

Poisson and negative binomial zero-altered models were constructed using the ‘hurdle’ function through the ‘pscl’ package in R^50^, and their comparison showed that negative binomial zero-altered (hereafter, ZANB) models performed better in all cases (i.e. dispersion parameter closer to one). ZANB models are two-part models, with a binomial part (hereafter binary part) modeling the probability that a zero value is observed and a count part modeling the non-zero data using a truncated negative binomial distribution^49^. This modeling approach enabled us to investigate two separate questions within a single statistical structure: what factors influenced whether violets were present in a plot (derived from the binary part of the model), and, if violets were present, what factors influenced the magnitude of their abundance (derived from the count part of the model).

In particular, to evaluate violet response to fire, we used Dataset1 and modeled violet presence and abundance through a ZANB model, using post-fire age, frequency of fire, season of fire (spring/winter), site and year as explanatory variables. To evaluate violet response to biotic and abiotic factors, we used Dataset2 and modeled violet presence and abundance through a ZANB model, using six biotic and two abiotic measurements as explanatory variables. For both ZANB models, we considered a global model (the m1 models in Tables S2 & S3) that would answer our ecological questions. We used variance inflation factors (VIF) to identify collinear explanatory variables that we removed from further analyses^48^. Collinearity was assessed with 5 as a cut-off value^51^. Then by minimizing the Akaike’s Information Criterion (AIC), we determined if the model could be simplified. Finally, model validation was undertaken by plotting residuals against fitted values^48^. To assess the significance of factor variables with more than two levels (i.e., “Site” and “Year” on Dataset1), we used the likelihood ratio tests between full and nested models. Subsequent pairwise comparisons for Site and Year differences were analyzed using Holm’s test with the family-wise error rate set at α = 0.05^52^ with the ‘glht’ function through the ‘multcomp’ package in R^53^.

The final model investigating the effect of fire history on violet abundance was fitted as:

$$\begin{array}{ccl}{\rm{Abundance}}\_{{\rm{fire}}}_{{\rm{i}}} & \sim  & {\rm{ZANB}}({{\rm{\mu }}}_{{\rm{i}}},{{\rm{\pi }}}_{{\rm{i}}},{\rm{k}})\\ E({\rm{Abundance}}\_{{\rm{fire}}}_{{\rm{i}}}) & = & \frac{1-{\pi }_{i}}{1-{P}_{0}}\ast \,{\mu }_{i}\,Where\,{P}_{0}={(\frac{k}{{\mu }_{i}+k})}^{k}\\ var({\rm{Abundance}}\_{{\rm{fire}}}_{{\rm{i}}}) & = & \frac{1-{\pi }_{i}}{1-{P}_{0}}\,\ast \,({\mu }_{i}+{{\mu }_{i}}^{2}+\frac{{{\mu }_{i}}^{2}}{k})-{(\frac{1-{\pi }_{i}}{1-{P}_{0}}\ast {\mu }_{i})}^{2}\\ Count\,part:\,log({\mu }_{i}) & = & Sit{e}_{i}+Yea{r}_{i}+Post\_fire\_ag{e}_{i}\\ Binary\,part:\,logit({\pi }_{\iota }) & = & Sit{e}_{i}+Yea{r}_{i}+Post\_fire\_ag{e}_{i}+Fire\_frequenc{y}_{i}\end{array}$$

where Abundance_fire_*i*_ is the number of violet individuals in plot *i* assuming a zero-altered negative binomial distribution with mean *μ*, probability *π* and dispersion *k*^48^.

The final model investigating the effect of biotic and abiotic environment on violet abundance was fitted as:

$$\begin{array}{ccl}{\rm{Abundance}}\_{{\rm{fire}}}_{{\rm{i}}} & \sim  & {\rm{ZANB}}({{\rm{\mu }}}_{{\rm{i}}},{{\rm{\pi }}}_{{\rm{i}}},{\rm{k}})\\ E({\rm{Abundance}}\_{{\rm{env}}}_{{\rm{i}}}) & = & \frac{1-{\pi }_{i}}{1-{P}_{0}}\ast \,{\mu }_{i}\,Where\,{P}_{0}={(\frac{k}{{\mu }_{i}+k})}^{k}\\ var({\rm{Abundance}}\_{{\rm{env}}}_{{\rm{i}}})\, & = & \frac{1-{\pi }_{i}}{1-{P}_{0}}\,\ast \,({\mu }_{i}+{{\mu }_{i}}^{2}+\frac{{{\mu }_{i}}^{2}}{k})-{(\frac{1-{\pi }_{i}}{1-{P}_{0}}\ast {\mu }_{i})}^{2}\\ Count\,part:log({\mu }_{i}) & = & Dead\_standing\_biomas{s}_{i}+Biocrus{t}_{i}+Roc{k}_{i}\\  &  & +\,Woody\_plant\_litte{r}_{i}\\ Binary\,part:logit({\pi }_{\iota }) & \,= & Dead\_standing\_biomas{s}_{i}+Biocrus{t}_{i}+Warm\_gras{s}_{i}\\  &  & +\,Cool\_gras{s}_{i}+Other\_vegetatio{n}_{i}+Bare\_groun{d}_{i}\\  &  & +\,Roc{k}_{i}+Woody\_plant\_litte{r}_{i}\end{array}$$

where Abundance_env_*i*_ is the number of violet individuals in plot *i* assuming a zero-altered negative binomial distribution with mean *μ*, probability *π* and dispersion *k*^48^.

### Statement of accordance

The authors declare that all the methods were carried out in accordance with the relevant guidelines and regulations.

## Results

### Violet response to prescribed fire

**Figure 1:**
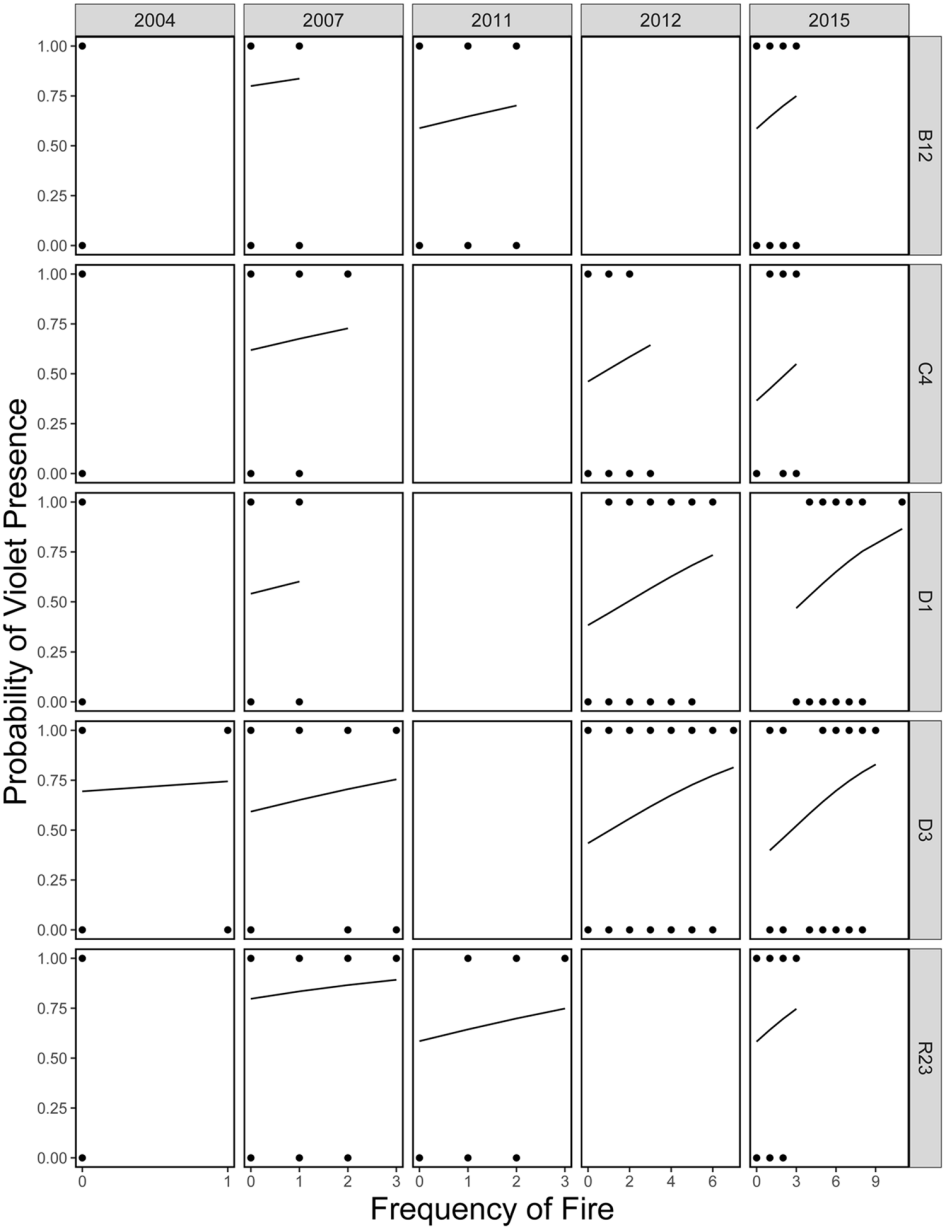
The effect of fire frequency on the likelihood of violet presence, with fitted regression lines taken from coefficients from the zero-altered part of the fire-history analysis. Sampling years (2004, 2007, 2011, 2012 and 2015) and study sites (B12, C4, D1, D3 and R23) are shown as distinct panels.

**Figure 2:**
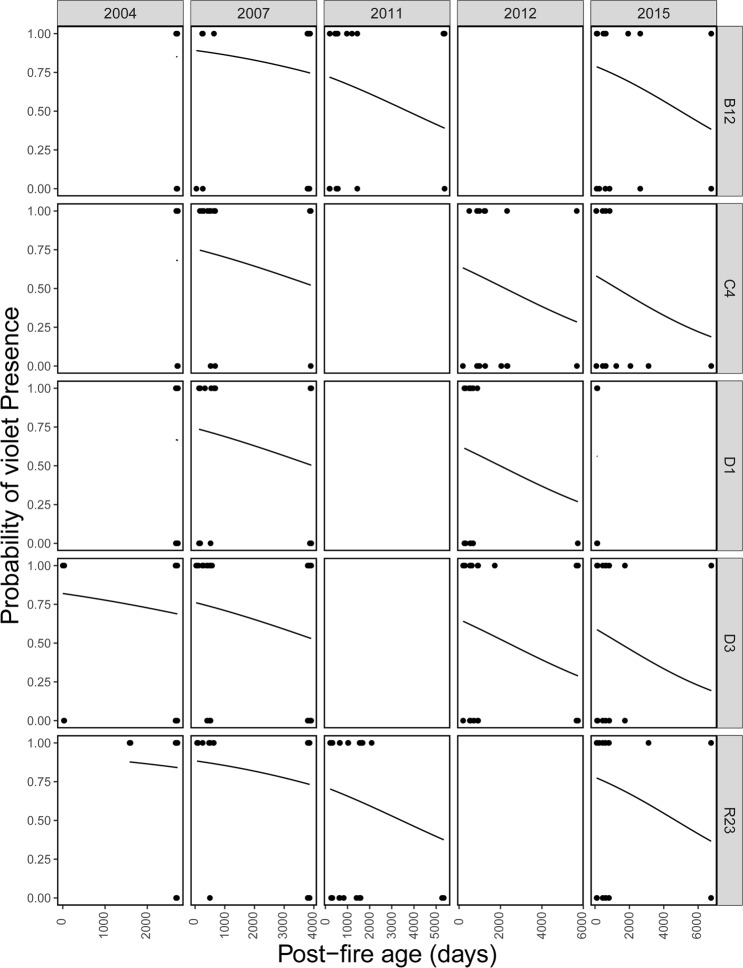
The effect of post-fire age on the likelihood of violet presence, with fitted regression lines taken from coefficients from the zero-altered part of the fire-history analysis. Sampling years (2004, 2007, 2011, 2012 and 2015) and study sites (B12, C4, D1, D3 and R23) are shown as distinct panels.

The frequency of prescribed fire was essential for determining the distribution of violets, with frequently burnt plots showing increased probability of violet presence (Fig. 1; Table 1, binary part). In addition, there was a marginally significant tendency (*P* = 0.056) for plots with high post-fire age to exhibit lower probability of violet presence (Fig. 2; Table 1, binary part). The overall effect of both “Site” and “Year” on violet presence (likelihood ratio test: *P* = 0.005 and *P* < 0.001 respectively) indicated a high temporal and spatial variation of violet distribution. The probability of violet presence was found to be higher during the early years of our study (2004, 2007 > 2011, 2012, 2015; pairwise comparisons: *P* < 0.05 in all cases), while sites R23 and B12 showed the highest probability of supporting violets (R23, B12 > D1, D3, C4; pairwise comparisons: *P* < 0.05 in all cases).

**Table 1:** Results of best-fit zero-altered negative binomial (ZANB) model testing the effects of prescribed fire on violet response. Dataset1 was used for this model.

	Parameter	Estimate	SE	*z*	*P*
*Count part (violet abundance)*	(Intercept)	−3.2400	0.5512	5.878	<0.001
	Site_C4	−0.6014	0.4054	−1.484	<0.138
	Site_D1	−0.7353	0.4583	−1.604	<0.109
	Site_D3	−0.8985	0.4084	−2.200	<0.028
	Site_R23	−0.5873	0.3670	−1.600	<0.110
	Year_2007	−0.3151	0.1829	−1.722	<0.085
	Year_2011	−0.4175	0.4031	−1.036	<0.300
	Year_2012	−0.2302	0.2555	−0.901	<0.367
	Year_2015	−0.9474	0.3047	−3.109	<0.002
	Post_fire_age	−0.0003	0.0001	−2.974	<0.003
*Binary part (violet presence-absence)*	(Intercept)	−2.3847	0.3964	−6.016	<0.001
	Site_C4	−1.0193	0.3142	−3.245	<0.001
	Site_D1	−1.2430	0.3049	−4.077	<0.001
	Site_D3	−1.0652	0.2915	−3.654	<0.001
	Site_R23	−0.0654	0.2760	−0.237	<0.813
	Year_2007	−0.4312	0.2213	−1.948	<0.051
	Year_2011	−1.6392	0.3605	−4.546	<0.001
	Year_2012	−1.1156	0.2742	−4.068	<0.001
	Year_2015	−1.5208	0.2997	−5.074	<0.001
	Post_fire_age	−0.0002	0.0001	−1.913	<0.056
	Fire_frequency	−0.1488	0.0687	−2.166	<0.030

The first level of the categorical variables “Site” (B12) and “Year” (2004) is used as an intercept (fixed reference against each of the remaining values).

**Figure 3:**
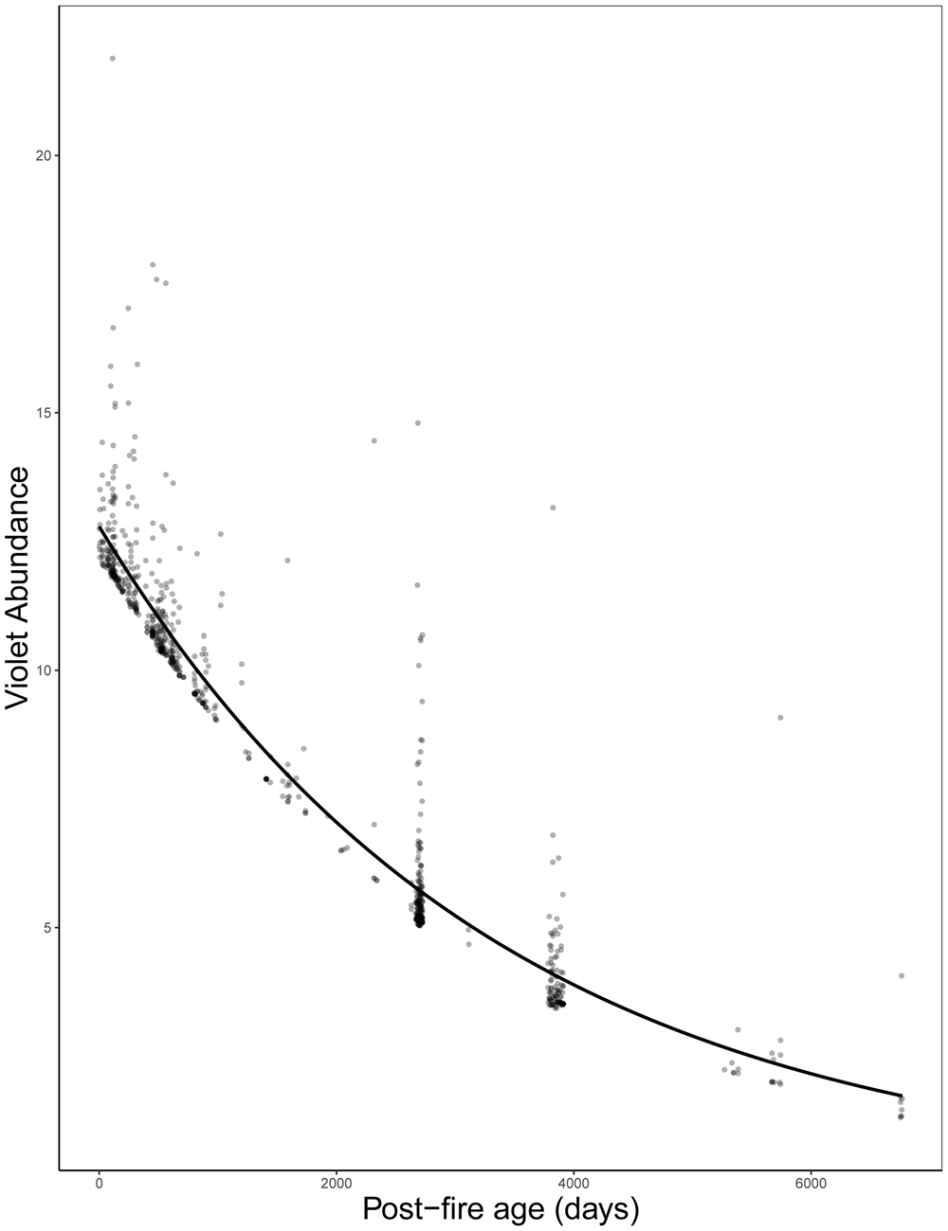
The effect of post-fire age on violet abundance, with fitted regression line from the negative binomial part of the fire-history analysis.

Where violets were present, violet abundance was not affected by prescribed fire frequency (model selection, Supplementary Table S2), but was negatively related to post-fire age (Fig. 3; Table 1, count part). Although there was a significant differentiation between D3 and B12 sites (Table 1, count part), the overall “Site” effect on the abundance of violets was non-significant (likelihood ratio test: *P* = 0.11). On the other hand, the year of sampling significantly affected violet abundance (likelihood ratio test: *P* = 0.041), with 2015 supporting, on average, higher violet abundance than 2004 (pairwise comparison: *P* = 0.014).

Overall, frequently burnt plots had a higher probability of supporting violets, while violets became more abundant in recently burnt areas and gradually declined with increasing post-fire age. Our results also revealed significant variation by site on the presence of violets. Our results further indicated that violets became more aggregated over time: in 2011, 2012 and 2015 they were present at fewer plots than in earlier sampling years, but in plots where they were present, they were at significantly higher abundances at the end of the study than at the beginning.

### Violet response to biotic and abiotic factors

Our results revealed a higher probability of violet presence on plots with more bare ground, and plots covered by greater amounts of warm-season grasses and biocrusts (Table 2, binary part). On the contrary, rockier plots and those covered with more dead standing biomass, cool-season grasses, and other vegetation presented a lower probability of supporting violets (Table 2, binary part). The amount of woody plant litter did not affect violet presence (Table 2, binary part).

**Table 2:** Results of best-fit zero-altered negative binomial (ZANB) model testing the effects of biotic and abiotic factors on violet response. Dataset2 was used for this model.

	Parameter	Estimate	SE	*z*	*P*
*Count part (violet abundance)*	(Intercept)	−3.230	0.128	−25.13	<0.001
	Dead_Standing_biomass	−0.033	0.005	−6.541	<0.001
	Biocrust	−0.047	0.024	−1.975	<0.050
	Rock	−0.029	0.011	−2.66	<0.008
	Woody plant litter	−0.050	0.015	−3.34	<0.001
*Binary part (violet presence-absence)*	(Intercept)	−0.495	0.533	−0.929	<0.353
	Dead_Standing_biomass	−0.028	0.007	−4.165	<0.001
	Biocrust	−0.157	0.054	−2.912	<0.004
	Warm_grass	−0.031	0.006	−4.857	<0.001
	Cool_grass	−0.015	0.007	−2.241	<0.025
	Other_vegetation	−0.012	0.004	−2.846	<0.004
	Bare_ground	−0.028	0.011	−2.605	<0.009
	Rock	−0.022	0.010	−2.127	<0.030
	Woody plant litter	−0.017	0.011	−1.577	<0.115

Where violets were present, the amount of dead standing biomass, biocrust, rock, and woody plant litter were the only habitat characteristics affecting violet abundance (model selection, Supplementary Table S3). Among plots where violets were present, plots with more dead standing biomass, rocks, and woody plant litter supported fewer violets, whereas plots with greater biocrust cover had more violets (Table 2, count part).

Overall, cool-season grasses and other vegetation were negatively associated with violet presence, while dead standing biomass and rocks were negatively associated with both probability of violet presence and violet abundances. Where violets were present, woody plant litter was negatively associated with violet abundance. In contrast, plots with more bare ground and those covered with warm-season grasses and biocrusts had a higher probability of supporting violets, while plots with more biocrust also had higher violet abundance.

## Discussion

Fire history strongly influenced violet presence and abundance. In particular, although we did not detect an effect of fire season on violets (model selection, Supplementary Table S2), we found that the probability of a plot to support violets increased with fire frequency (Fig. 1). Even at the most frequently burnt plots (i.e. burned 11 times in a 12-year period), our results indicate that even near-annual burning is still positively associated with violet presence. Further, plots with a low post-fire age (i.e., burned recently) had a marginally significant tendency to support violets (Fig. 2), while, when violets were present, low post-fire age plots supported the highest violet abundances (Fig. 3). These results are consistent with the findings of Latham *et al*.^43^, which showed greater violet density in burnt versus unburnt areas. Our extensive dataset and rigorous analytical approach, however, extended these results to a gradient of fire history and distinguished the effects of different components of fire history (burn frequency and post-burn duration) on both the presence and abundance of violets. Such results provide new insights by demonstrating that repeated fires have cumulative positive effects on violets and that the positive effects of fire for violets diminish with time since last fire.

Our results suggest a management strategy including frequent prescribed fires and small inter-fire intervals may maintain high prevalence and abundance of violets across the landscape. Managers must consider the needs of other species of conservation concern and balance other management goals when determining the appropriate fire interval for grassland habitats. Given the exclusive reliance of *S. i. idalia* on violets as host plants, however, at sites where violet propagules are not limiting, such a management strategy with frequent fires and small inter-fire intervals may be highly effective in preparing unmanaged grassland for regal reintroduction efforts. The same management strategy should also translate into increased *S. i. idalia* abundances in its currently occupied habitat as well, though we caution that prescribed fire may also cause direct mortality if burning takes place during immature life stages when they cannot escape incoming flames^27^. The relative importance of direct effects (that cause mortality of immature butterfly stages) versus indirect effects (that affect butterflies via changes to host plants, nectar plants, and vegetation structure and composition) of fire on butterfly populations is almost unknown (but see^54^), but the potential for this trade-off suggests that avoiding burns during the immature life stages of regals and/or maintaining a mosaic landscape with a spatially and temporally heterogeneous fire history that continually includes unburnt refugia is important^55^.

Although violet abundances did not differ by site in plots where they were present, the prevalence of violets varied spatially, suggesting patchy distribution patterns of violets in some sites (D1, D3, and C4) and more homogeneous distributions in others (R23 and B12). These spatially variable patterns, which were evident even when controlling for fire history, may be explained by differences in topography or microclimate. Another possibility could be site differences in fuel availability, resulting in spatial variation in fire characteristics^56^. Indeed, fire characteristics such as fire severity have been linked to plant functional trait filtering and thus to shaping patterns of community assembly^57^. In general, prescribed fire, alone or interacting with other disturbances, can induce changes in community composition, seed production and/or functional traits^58–60^, potentially affecting dispersal-based processes and consequently community assembly^61^. Further, consistent with the response-effect framework proposed by Lavorel and Garnier^62^, shifts in violet functional traits emerging from heterogeneous prescribed fires may affect the distribution patterns of *S. i. idalia* host plants and larval-host plant interactions (e.g. via shifts in leaf palatability).

We also detected temporal variation in violet distribution and abundance. Specifically, violets were present at more plots during earlier years (2004 and 2007) but had higher abundances at the end (2015) than at the beginning (2004) of the survey, suggesting increasing aggregation over time. The causes of such aggregation over time, and the among-site differences patchiness remain unclear, but one possibility is that small-scale variation in fire severity led to within-site heterogeneity in disturbance of the existing plant community, changing competitive regimes and conditions for violet growth. Fire effects on biotic interactions could provide an alternate pathway: for instance, because violets are often dependent on ants for seed dispersal^29^, the ongoing aggregation of violet populations in our system might be explained by the effects of prescribed fire on myrmecochory through changes in ant distributions or population dynamics^63^. The observed changes in violet distribution could have negative effects on the *S. i. idalia* population: because females use a “sweepstakes” oviposition strategy^64^, increasing aggregation and local patchiness of violets could lead to wasted reproductive effort and reduced recruitment as larvae hatch in sites where their host plants are absent.

Our second analysis also identified biotic and abiotic characteristics associated with violet presence and abundance. Specifically, our results indicated that within grasslands, openings dominated by warm-season grasses and biocrust communities have a high probability of supporting violets. In contrast, violets were rarely found in rocky plots and those mostly covered by dead standing biomass, cool-season grasses and other vegetation. When violets were present, biocrusts were positively associated with violet abundance, while rocky habitats and those covered by dead standing biomass and woody plant litter were negatively associated with violet abundance.

These associations may be explained in relation to the ecology and natural history of violets. The positive association of bare ground and negative association of plant cover (‘other vegetation’ in our model) with violet presence suggests violets may benefit from the direct light and lack of competition with other plants in such areas, or that the germination of violet seeds may benefit from the warm-dry conditions^65^ that bare ground provides for longer periods than areas with plant cover. However, Latham *et al*.^43^ showed that fire increases both violet abundance and bare ground cover at the same time, and thus the positive effect of bare-ground captured by our analysis might be a signal of the positive effect of fire on both bare-ground and violet distribution. Rocky areas may also provide direct light, lowered plant competition, and warm-dry conditions, but the negative associations of rocky ground and violets suggest rocky areas do not provide suitable substrate for growth. Apart from occupying space otherwise potentially available for violet establishment, dead standing plant biomass probably does not have a direct negative effect on violet establishment. However, increased cover of dead standing plant biomass and of woody plant litter is indicative of habitats experiencing long periods of fire suppression, which, according to our first model, decreases habitat suitability for violet establishment. Additionally, although grasses may regrow quickly after a fire, the more negative effect of cool-season grasses on violet distribution than warm-season grasses may be explained by higher productivity of cool-season grasses at low temperatures, allowing growth early in the spring^66^. Such early growth may reduce the light quantity and quality reaching violet plants (which are <15 cm tall) and seeds during its primary period of aboveground growth^67^.

In addition, our results indicate that biocrust cover has a positive impact on both violet distribution and abundance. Biocrust communities have been shown to modulate soil hydrological processes^68^ thus affecting microclimate, with biocrust-dominated habitats being very efficient in retaining water under intense rainfalls and drying faster during dry periods^69^. Given that several studies have provided evidence that stratification promotes violet germination^70–73^, it is possible that the microenvironment created by biocrusts might facilitate violet germination, by providing warm-dry conditions during violet seed dispersal in summer, followed by stratification (cold-wet conditions). In addition, biocrust-forming mosses create structures that can easily trap and hold seeds and thus moss-dominated biocrusts may facilitate violet recruitment by seed retention. In the same sense, Freestone^74^ previously showed that a moss species (*Didymodon tophaceus*) facilitated the recruitment of *Delphiniium uliginosum*, a rare species specializing in serpentine wetlands. Finally^75^, showed that biocrust cover significantly influences richness and abundance of ant communities, and thus a potential indirect effect of biocrust cover on violet distribution and abundance through changes in myrmecochory is possible.

The importance of biocrusts for violets has several implications for management. First, although heavy vehicle military training may result in soil degradation, oftentimes less impactful military disturbance may have positive impact on biodiversity and especially on biocrust formation^76,77^. Thus, as long as heavy military vehicle maneuvers are avoided, military training and the maintenance of viable host plant populations may be able to coexist. Second, although high-intensity fires may reduce the abundance of biocrust organisms^78^, low-intensity fires have only mild direct effects on biocrust composition^79^. In addition, relatively frequent fires (biennial) have been shown to facilitate biocrust communities (i.e. increase diversity and abundance of biocrust species) by reducing the abundance and cover of vascular plants competing for the same resources^80^. Consequently, our results indicate that the use of low-intensity and relatively frequent prescribed fires should maintain biocrust-dominated habitats, which are associated with the most productive violet populations in our system. Finally, even when the fire history of a site is unknown, the presence of high amounts of biocrust within a grassland context may signal promising candidate sites for violet and *S. i. idalia* reintroduction.

## Conclusions

Conserving a specialized plant-insect interaction is a multifactorial process, and maintaining abundant host plant populations is a highly important part of it^81,82^. This study sheds light on the mechanisms through which prescribed fire management affects the distribution and abundance of host plants (*Viola spp*.) of the last viable population of an extremely rare butterfly, the eastern regal fritillary (*S. i. idalia*), and provides a roadmap for efficient site selection for restoration and reintroduction actions. We showed that frequent prescribed fire is highly efficient in increasing violet distribution, while using small intervals between prescribed fires efficiently maintains high levels of violet abundance. However, given that prescribed fire may also cause direct mortality to *S. i. idalia*, special care must be taken in avoiding prescribed fire application during low-mobility periods (as suggested by Zografou *et al*.^15^). Although prescribed fire in FIG-NGTC is not primarily undertaken for the maintenance of viable hostplant populations for *S. i. idalia*, our results indicate that during the 12-year study period violets became significantly more abundant. However, at the same time we recorded a shift towards more aggregated and patchy violet populations, indicating a potential indirect effect of prescribed fire on violet dispersal, possibly through changes in myrmecochory. Consequently, to provide more detailed post-fire interval suggestions, potential direct effects of prescribed fire on butterfly and ant communities should be investigated.

In addition, consistent with guidance from Henderson *et al*.^21^, that identifying, creating and maintaining high quality habitats is the most impactful way to sustain regal populations, we determined habitat characteristics well correlated with violet host plants. Specifically, we found that habitats with openings, dominated by warm-season grasses and biocrust communities are the most suitable ones for supporting violets, while increased cover of biocrust communities is indicative of the most abundant violet populations. Hence, management actions favoring these habitat characteristics should facilitate the establishment of new violet populations or increase existing ones, and should increase the effectiveness of management and reintroduction efforts for violets and eastern regal fritillary butterflies. Finally, according to our results, the application of frequent prescribed fire and the avoidance of high-intensity fires and heavy military vehicle training is recommended to shape and maintain suitable habitats that support widespread and abundant violet populations. More broadly, our results suggest management approaches that may effectively accommodate both conservation of *S. i. idalia* and human activities at FIG-NGTC, and that could be replicated in other fire-managed areas and for other butterfly and host plant species.

## Supplementary information

Supplementary information

## Data Availability

The datasets analyzed during the current study are available from the corresponding author on reasonable request.
