# Supplementary material for: Prescribed fire maintains host plants of a rare grassland butterfly
Source: Sci Rep. 2019 Nov 14;9:16826. doi: 10.1038/s41598-019-53400-1 (PMC6856363; doi:10.1038/s41598-019-53400-1)
Supplement: Supplementary file 1 — Supplementary information [file 41598_2019_53400_MOESM1_ESM.pdf]

Prescribed fire maintains host plants of a rare grassland butterfly

George C. Adamidis[1], Mark T. Swartz[2], Konstantina Zografou[1], Brent J. Sewall[1]

[1]Department of Biology, 1900 North 12th St., Temple University, Philadelphia, PA 19122, USA

[2]The Pennsylvania Department of Military and Veterans Affairs, Fort Indiantown Gap National Guard Training Center, Annville, PA 17003, USA

Correspondence and requests for materials should be addressed to G.C.A. (email: adamidis@env.aegean.gr)

Table S1. Coordinates of the study sites within grassland habitats at Fort Indiantown Gap National Guard Training Center, USA.

No.	Site	Latitude	Longitude
1	B12	40°27'0.191"	-76°37'3.086"
2	C4	40°26'51.549"	-76°38'1.542"
3	D1	40°25'11.815"	-76°42'28.509"
4	D3	40°24'55.502"	-76°42'37.163"
5	R23	40°26'11.426"	-76°39'9.98"

Table S2. Model selection criteria and parameter estimates among competing zero-altered negative binomial models predicting violet response to prescribed fire.

Model	Model ID	df	AIC	Delta AIC (Δi)	Akaike weight (wi)
**~ Site + Year + Post_fire_age | Site + Year + Post_fire_age + Fire_frequency**	m4	22	5518.02	0	0.50
~ Site + Year + Post_fire_age + Fire_frequency | Site + Year + Post_fire_age + Fire_frequency + Fire_season	m2	24	5519.45	1.43	0.25
~ Site + Year + Post_fire_age + Fire_frequency | Site + Year + Post_fire_age + Fire_frequency	m3	23	5519.99	1.97	0.19
~ Site + Year + Post_fire_age + Fire_frequency + Fire_season | Site + Year + Post_fire_age + Fire_frequency + Fire_season	m1	26	5522.46	4.44	0.06

The lowest Akaike Information Criterion (AIC) is subtracted from each model AIC to obtain the ΔAIC (Δi), which is then used to calculate the relative likelihood or weight (wi) of each model. In all models, the count part is presented before the binary part. Final model is indicated by bold text.

Table S3. Model selection criteria and parameter estimates among competing zero-altered negative binomial models predicting violet response to biotic and abiotic factors.

Model	Model ID	df	AIC	Delta AIC (Δi)	Akaike weight (wi)
**~ Dead_standing_biomass + Biocrust + Rock + Woody_plant_litter | Dead_standing_biomass + Biocrust + Warm_grass + Cool_grass + Other_vegetation + Bare_ground + Rock + Woody_plant_litter**	m5	15	4103.23	0	0.29
~ Dead_standing_biomass + Biocrust + Rock + Woody_plant_litter | Dead_standing_biomass + Biocrust + Warm_grass + Cool_grass + Other_vegetation + Bare_ground + Rock	m6	14	4104.07	0.84	0.19
~ Dead_standing_biomass + Biocrust + Other_vegetation + Rock + Woody_plant_litter | Dead_standing_biomass + Biocrust + Warm_grass + Cool_grass + Other_vegetation + Bare_ground + Rock + Woody_plant_litter	m4	16	4104.85	1.62	0.13
~ Dead_standing_biomass + Biocrust + Rock + Woody_plant_litter | Dead_standing_biomass + Biocrust + Warm_grass + Other_vegetation + Bare_ground	m8	12	4105.47	2.24	0.09
~ Dead_standing_biomass + Biocrust + Warm_grass + Other_vegetation + Rock + Woody_plant_litter | Dead_standing_biomass + Biocrust + Warm_grass + Cool_grass + Other_vegetation + Bare_ground + Rock + Woody_plant_litter	m3	17	4105.64	2.41	0.09
~ Dead_standing_biomass + Biocrust + Rock + Woody_plant_litter | Dead_standing_biomass + Biocrust + Warm_grass + Cool_grass + Other_vegetation + Bare_ground	m7	13	4105.72	2.49	0.08
~ Dead_standing_biomass + Biocrust + Rock + Woody_plant_litter | Dead_standing_biomass + Biocrust + Warm_grass + Bare_ground	m9	11	4105.73	2.5	0.08
~ Dead_standing_biomass + Biocrust + Warm_grass + Other_vegetation + Bare_ground + Rock + Woody_plant_litter | Dead_standing_biomass + Biocrust + Warm_grass + Cool_grass + Other_vegetation + Bare_ground + Rock + Woody_plant_litter	m2	18	4107.59	4.36	0.03
~ Dead_standing_biomass + Biocrust + Warm_grass + Cool_grass + Other_vegetation + Bare_ground + Rock + Woody_plant_litter | Dead_standing_biomass + Biocrust + Warm_grass + Cool_grass + Other_vegetation + Bare_ground + Rock + Woody_plant_litter	m1	19	4109.56	6.33	0.01

The lowest Akaike Information Criterion (AIC) is subtracted from each model AIC to obtain the ΔAIC (Δ_i), which is then used to calculate the relative likelihood or weight (w_i) of each model. In all models, the count part is presented before the binary part. Final model is indicated by bold text.
